# TRAF5 and TRAF3IP2 Gene Polymorphisms Are Associated with Behçet's Disease and Vogt-Koyanagi-Harada Syndrome: A Case-Control Study

**DOI:** 10.1371/journal.pone.0084214

**Published:** 2014-01-08

**Authors:** Qin Xiang, Lu Chen, Shengping Hou, Jing Fang, Yan Zhou, Lin Bai, Yunjia Liu, Aize Kijlstra, Peizeng Yang

**Affiliations:** 1 Chongqing Key Laboratory of Ophthalmology and Chongqing Eye Institute; The First Affiliated Hospital of Chongqing Medical University, Chongqing, P. R. China; 2 University Eye Clinic Maastricht, Maastricht, The Netherlands; Kunming Institute of Zoology, Chinese Academy of Sciences, China

## Abstract

**Background:**

TRAF5 and TRAF3IP2 have been reported to be associated with several autoimmune diseases. Behçet's disease (BD) and Vogt-Koyanagi-Harada (VKH) syndrome are two autoimmune uveitis entities whereby both genetic and environmental factors are thought to be involved.

**Objective:**

The role of TRAF5 and TRAF3IP2 in BD and VKH has not yet been reported and was therefore the subject of this study.

**Methods:**

The study included 789 BD patients, 940 VKH patients and 1601 healthy unrelated individuals. Genotyping was performed by polymerase chain reaction-restriction fragment length polymorphism (PCR-RFLP) or TaqMan® SNP Genotyping Assay. Real-Time PCR was used to detect mRNA expression from PBMCs obtained from healthy controls with (n = 22) or without (n = 79) stimulation. Levels of TNF-α, IL-6 and IL-8 in culture supernatants were measured by ELISA (n = 22).

**Results:**

Three SNPs (rs6540679, rs12569232, rs10863888) of TRAF5 and rs13210247 of TRAF3IP2 were significantly associated with Behçet's disease and VKH syndrome (corrected P values ranging from 9.45×10^−12^ to 0.027). TRAF3IP2 rs33980500 and rs13190932 were not polymorphic in Han Chinese. Following stimulation by lipopolysaccharide (LPS), carriers of the GG genotype of rs6540679/TRAF5 had a higher TRAF5 mRNA expression (p = 0.004) and an increased TNF-α (p = 0.0052) and IL-6 (p = 0.0014) level compared with AA and AG genotype carriers.

**Conclusion:**

This study provides evidence that TRAF5 and TRAF3IP2 genes are involved in the development of BD and VKH syndrome. Functional research suggested that TRAF5 gene polymorphisms may regulate TRAF5 expression and downstream inflammatory cytokines such as TNF-α and IL-6.

## Introduction

Uveitis is an intraocular inflammation that is caused by either infectious or noninfectious mechanisms and which can lead to serious visual impairment. To unravel the immunological mechanisms that cause uveitis we have focused on two commonly occurring uveitis entities in China, namely Behcet's disease (BD) and Vogt Koyanagi Harada (VKH) syndrome [1], [2].

BD is an autoinflammatory disease characterized by a diverse spectrum of clinical manifestations including recurrent oral aphthae, uveitis, multiform skin lesions and genital ulceration [3], [4]. It has a relatively high prevalence along the Silk Road countries such as Japan, China, Turkey, and the Mediterranean region [5]. The usual onset is between the age of twenty to forty. The cause of BD is still unknown but epidemiological studies have suggested that certain genetic factors play an important role in its development [6]. We and others have recently identified polymorphisms in multiple immunoregulatory genes as a risk factor for developing BD, such as CD40, STAT3, STAT4 and JAK2 [7], [8], [9]. These pathways are extremely complex and many other gene polymorphisms remain to be studied to unravel the exact genetic susceptibility to BD.

VKH syndrome is a multisystem autoimmune disorder directed against melanocytes and is characterized by bilateral granulomatous uveitis with meningeal irritation sign (MIS), dysacusis, vitiligo, poliosis and alopecia [10]. It is one of the most common uveitis entities in Chinese as well as in individuals of American-Indian descent. VKH has been shown to be associated with certain HLA genes [11], [12]. However, this association cannot explain the whole genetic risk for VKH and further research is needed to identify potential non-HLA susceptibility genes. We have recently studied polymorphisms of various immune response genes in the pathogenesis of VKH syndrome and showed associations with several non HLA genes [13], [14], [15].

In our search for a possible role of as yet not identified polymorphisms in immunoregulatory genes in the pathogenesis of uveitis, we assessed factors that have been shown to play a role in autoimmune diseases that share features with uveitis. One of the polymorphic genes that were recently shown to be involved in autoimmune disease such as inflammatory bowel disease, rheumatoid arthritis and diabetes belong to the so called family of Tumor necrosis factor (TNF)-receptor-associated factors (TRAF).

TRAF5 is one of the factors belonging to the TRAF family and is significantly expressed in lung, thymus, spleen and kidney as well as in peripheral blood cells [16]. It is an adapter protein and signal transducer that links members of the tumor necrosis factor (TNF) receptor family to different signaling pathways and plays a critical role in the activation of NF-kappa-B leading to the expression of various inflammatory cytokines [17]. It has been demonstrated that TRAF5 polymorphisms are associated with susceptibility to rheumatoid arthritis (RA) [18].

TRAF3IP2 is another member of the TRAF family and encodes Act1 protein, which binds to TRAF5 thereby playing an important role in controlling inflammatory responses and autoimmunity through its effect on CD40L and IL-17 signaling [19], [20], [21]. Mutations of the TRAF3IP2 gene have been associated with susceptibility to several autoimmune diseases, such as psoriasis, psoriatic arthritis, chronic plaque psoriasis, inflammatory bowel disease and type 1 diabetes [22], [23], [24], [25], [26]. Of interest are the observations that IL-17 can induce Act1-TRAF2-TRAF5 complex formation leading to a downstream inflammatory response [20], [27]. Earlier studies showed that Il-17 plays an important role in the pathogenesis of both BD and VKH [28], [29] , providing support for a further study into a possible role of TRAF gene polymorphisms in uveitis.

**Figure 1:**
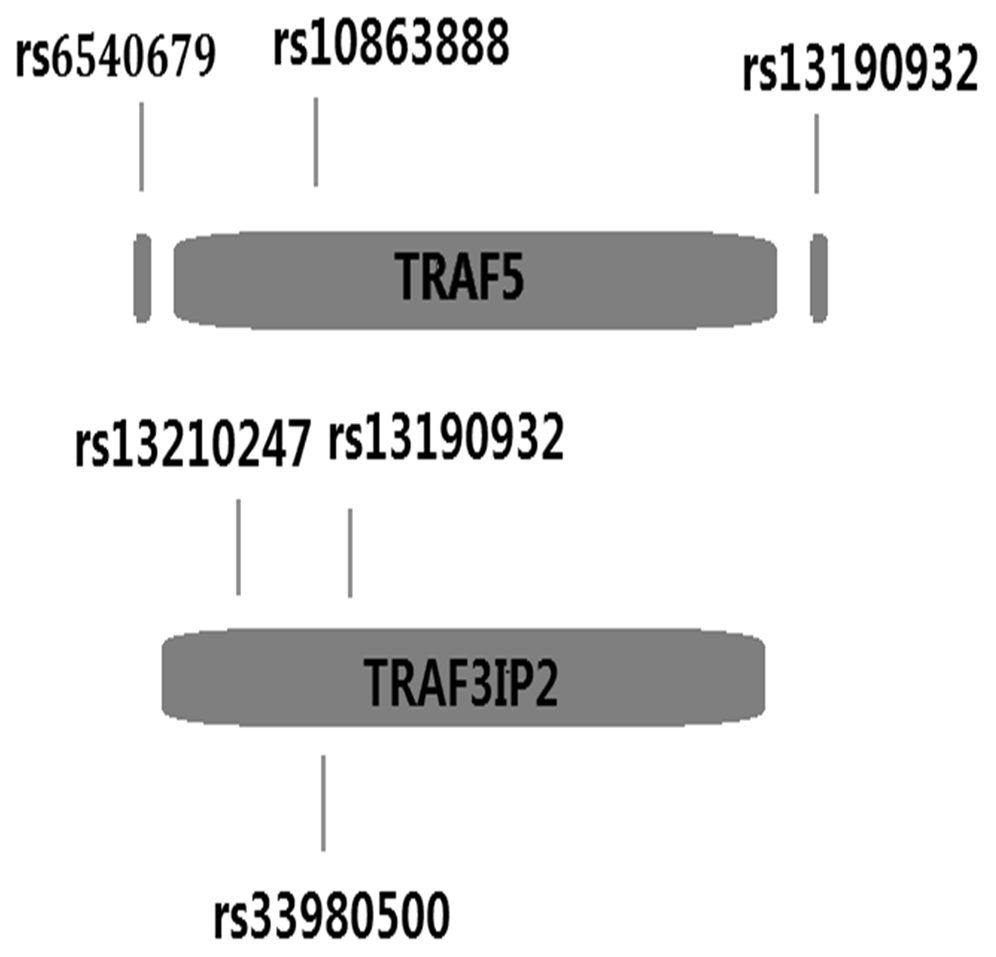
Schematic location of the chosen SNPs of TRAF5 and TRAF3IP2.

Based on earlier reports in RA, psoriasis and psoriatic arthritis [18], [23], [24], [25] and linkage disequilibrium patterns in Han Chinese, in combination with Japanese data in the Hap Map database, we selected three SNPs (rs6540679 Chr.1: 211491152, rs12569232 Chr.1: 211553064, rs10863888 Chr.1: 211502769) of TRAF5 and three SNPs (rs13210247 Chr.6: 111922720, rs33980500 Chr.6: 111913262, rs13190932 Chr.6: 111913070) of TRAF3IP2 as candidate SNPs for our study (Figure 1).

## Methods

### Patients and healthy controls study population

All subjects gave their written informed consent for this study, and the study protocol was approved by the Ethics Committee of the First Affiliated Hospital of Chongqing Medical University, Chongqing, China (Permit Number: 2009-201008). All procedures followed the tenets of the Declaration of Helsinki.

A case-control genetic study of two genes in individuals with or without uveitis was performed. 789 BD patients, 940 VKH patients and 1601 healthy unrelated individuals were included in this study. All subjects were of Han Chinese descent. Blood samples were obtained from the First Affiliated Hospital of Chongqing Medical University (Chongqing, China) and the Uveitis Study Center of the Sun Yat-sen University (Guangzhou, China) through 2005 until 2013.

The diagnosis of BD was based on the criteria of the International Study [30]. The clinical characteristics of the BD patients were assessed at the time of diagnosis and are summarized in Table S1. The diagnosis of VKH syndrome fulfilled the revised criteria for this disease [10]. The clinical characteristics of the patients are presented in Table S2.

### DNA extraction and genotyping

Genomic DNA samples of BD, VKH patients and healthy controls were extracted by using the QIAamp DNA Blood Mini Kit (Qiagen, Valencia, CA). Rs6540679, rs33980500, rs13210247, rs13190932 were genotyped by polymerase chain reaction-restriction fragment length polymorphism (PCR-RFLP), the primers used to amplify the target DNA sequence by PCR are shown in Table S3. Digestion products were visualized on a 4% agarose gel and stained with GoldView™ (SBS Genetech, Beijing, China). Rs12569232 (TagMan assay ID: C_26176827_20) and rs10863888 (TagMan assay ID: C_26176858_10) genotypes were investigated using the TaqMan® SNP Genotyping Assay (Applied Biosystems, Foster City, CA, USA) on the Applied Biosystems 7500 Real-Time PCR system. All the operations were performed according to manufacturers' instructions. The analysis was performed using TaqMan® Genotyper Software. Direct sequencing was also performed by the Beijing Biomed Co.,Ltd (Beijing, China) using randomly selected subjects (10% of all samples) to validate the accuracy of genotyping. The genotyping success rate of the various SNPs ranged between 93.6% and 100%.

### Real-time PCR

Peripheral blood mononuclear cells (PBMCs) were prepared from heparinized blood by Ficoll-Hypaque density-gradient centrifugation. Isolated PBMCs were cultured in 24-well plates containing RPMI-1640 supplemented with 10% calf serum (Greiner, Wemmel, Belgium), 2 mM L-glutamine and 100 U/ml penicillin/streptomycin (Invitrogen, Carlsbad, CA). Cells were left unstimulated or were stimulated with a cocktail of anti-CD3 (5 µg/ml, eBioscience, San Diego, CA, USA) and anti-CD28 antibodies (5 µg/ml, eBioscience, San Diego, CA, USA) to mimic antigen presentation. Alternatively the cells were stimulated with LPS (1 ng/ml, Sigma-Aldrich Co. LLC), at 37°C in humidified 5% CO_2_ for 72 hours at a density of 1×10^6^ cells/ml. Total RNA was extracted from the cultured PBMCs using TRIzol (Invitrogen), followed by reverse transcription using a transcriptase kit (Takara Biotechnology Co. Ltd., Dalian, China.). Real-time quantitative PCR was performed to compare the mRNA expression of TRAF3IP2 and TRAF5 gene, using the Applied Biosystem 7500 Real-time PCR System and was determined using the SYBR Green I Assay kit (Applied Biosysterms) and normalized to β-actin mRNA. Relative expression levels were calculated using the 2^−ΔΔCt^ method. The sense and antisense primers used in this experiment are depicted in Table S4.

### Cytokine Measurements

The concentration of IL-8, IL-6 and TNF-α in cell culture supernatants was measured with the human Duoset enzyme-linked immunosorbent assay (ELISA) Development kit (R&D System, Minneapolis, MN) according to the manufacturers' instructions.

### Statistical analysis

Hardy-Weinberg equilibrium was tested using the χ2 test for goodness of fit and a p-value<0.05 was considered as a significant disequilibrium. The patterns of linkage disequilibrium (LD) of the tested SNPs were compared using Haploview (version 4.0, Broad Institute of MIT and Harvard, Cambridge, MA). Allele and genotype frequencies were compared between patients and controls by the χ2 test or two-sided Fisher' exact test using SPSS (version 13.0; SPSS Inc, Chicago, IL). The p values were corrected (p_c_) with the Bonferroni correction by multiplying with the number of analyses performed. For Table 1 and Table 2, p_c_ equaled to a p value multiplied by 9 (for genotype analysis) or 3 (for allele analysis). A p _c_<0.05 was considered significant.

**Table 1:** Polymorphisms of the TRAF5 and TRAF3IP2 genes in ocular Behçet's Disease.

Gene	SNPs	Genotype	BD n (%)	Control	P value	Pc value	OR (95%CI)
		Allele	n (%)	n (%)			
TRAF5	rs10863888	AA	74 (10)	170 (10.8)	0.566	5.094	0.919 (0.689–1.226)
		AG	359 (48.6)	650 (41.3)	0.001	0.009	1.343 (1.127–1.601)
		GG	306 (41.4)	754 (47.9)	0.003	0.027	0.769 (0.644–0.917)
		A	507 (34.3)	990 (31.4)	0.053	0.477	1.138 (0.998–1.298)
		G	971 (65.7)	2158(68.6)	0.053	0.477	0.879 (0.771–1.002)
	rs12569232	CC	9 (1.2)	20 (1.2)	0.893	8.037	0.947 (0.429–2.090)
		CG	96 (12.6)	304 (19.0)	1.20×10^−4^	1.08×10^−3^	0.617 (0.481–0.790)
		GG	655 (86.2)	1277(79.8)	1.56×10^−4^	1.40×10^−3^	1.583 (1.246–2.011)
		C	114 (7.5)	344 (10.7)	4.34×10^−4^	1.30×10^−3^	0.674 (0.540–0.840)
		G	1406(92.5)	2858(89.3)	4.34×10^−4^	1.30×10^−3^	1.484 (1.190–1.852)
	rs6540679	AA	58 (7.4)	81 (5.2)	0.036	0.324	1.449 (1.022–2.053)
		AG	355 (45.0)	497 (31.9)	4.00×10^−10^	3.60×10^−9^	1.750 (1.467–2.086)
		GG	376 (47.7)	982 (62.9)	1.35×10^−12^	1.22×10^−11^	0.536 (0.451–0.637)
		A	471 (29.8)	659 (21.1)	3.86×10^−11^	1.16×10^−10^	1.589 (1.384–1.824)
		G	1107(70.2)	2461(78.9)	3.86×10^−11^	1.16×10^−10^	0.629 (0.548–0.722)
TRAF3IP2	rs13210247	AA	539 (88.4)	1513(95.1)	1.79×10^−8^	1.61×10^−7^	0.391 (0.280–0.548)
		AG	71 (11.6)	76 (4.8)	7.83×10^−9^	7.05×10^−8^	2.626 (1.873–3.682)
		GG	0 (0.0)	2 (0.1)	0	0	
		A	1149(94.2)	3102(97.5)	6.91×10^−8^	2.07×10^−7^	0.417 (0.301–0.579)
		G	71 (5.8)	80 (2.5)	6.91×10^−8^	2.07×10^−7^	2.396 (1.728–3.322)

SNP, single-nucleotide polymorphism; VKH, Vogt-Koyanagi-Harada; BD, Behçet's disease; OR, odds ratio; CI, confidence interval; Pc, Bonferroni corrected p value which p_c_ equaled to a p value multiplied by 9 (for genotype) or 3 (for allele).

**Table 2:** Polymorphisms of the TRAF5 and TRAF3IP2 genes in VKH syndrome.

Gene	SNPs	Genotype	VKH	Control	P value	Pc value	OR (95%CI)
		Allele	n (%)	n (%)			
TRAF5	rs10863888	AA	94 (10.4)	170 (10.8)	0.734	6.606	0.955 (0.732–1.246)
		AG	416 (45.9)	650 (41.3)	0.027	0.243	1.204 (1.022–1.420)
		GG	397 (43.8)	754 (47.9)	0.047	0.423	0.847 (0.718–0.998)
		A	604 (33.3)	990 (31.4)	0.179	1.611	1.088 (0.962–1.231)
		G	1210 (66.7)	2158(68.6)	0.179	1.611	0.919 (0.812–1.040)
	rs12569232	CC	3 (0.3)	20 (1.2)	0.017	0.153	0.254 (0.075–0.857)
		CG	89 (9.5)	304 (19.0)	1.48×10^−10^	1.33×10^−9^	0.446 (0.347–0.574)
		GG	848 (90.2)	1277(79.8)	6.28×10^−12^	5.65×10^−11^	2.339 (1.826–2.995)
		C	95 (5.1)	344 (10.7)	3.15×10^−12^	9.45×10^−12^	0.442 (0.350–0.559)
		G	1785 (94.9)	2858(89.3)	3.15×10^−12^	9.45×10^−12^	2.262 (1.788–2.860)
	rs6540679	AA	20 (2.2)	81 (5.2)	2.90×10^−4^	2.61×10^−3^	0.411 (0.250–0.675)
		AG	284 (31.2)	497 (31.9)	0.751	6.759	0.972 (0.815–1.159)
		GG	605 (66.6)	982 (62.9)	0.071	0.639	1.171 (0.986–1.391)
		A	324 (17.8)	659 (21.1)	0.005	0.015	0.810 (0.699–0.939)
		G	1494 (82.2)	2461(78.9)	0.005	0.015	1.235 (1.065–1.431)
TRAF3IP2	rs13210247	AA	649 (90.0)	1513(95.1)	4.28×10^−6^	3.85×10^−5^	0.465 (0.333–0.648)
		AG	71 (9.8)	76 (4.8)	3.68×10^−6^	3.31×10^−5^	2.177 (1.556–3.048)
		GG	1 (0.1)	2 (0.1)	1.000	9.000	1.103 (0.100–12.189)
		A	1369 (94.9)	3102(97.5)	7.19×10^−6^	2.16×10^−5^	0.484 (0.350–0.668)
		G	73 (5.1)	80 (2.5)	7.19×10^−6^	2.16×10^−5^	2.068 (1.496–2.858)

## Results

Six SNPs were successfully genotyped and confirmed to Hardy-Weinberg expectation in controls. A linkage disequilibrium analysis using Haploview software showed that the selected SNPs were not linked. Both rs33980500 and rs13190932 of the TRAF3IP2 gene were not polymorphic in Han Chinese and were therefore not analyzed further. Analysis of the clinical symptoms of our BD patients revealed six primary features, including oral ulcer (100.0%), skin lesions (78.0%), genital ulcer (57.9%), arthritis (39.1%), positive pathergy test (24.5%) and hypopyon (22.3%). The VKH patients could be subdivided according to seven clinical features, including nuchal rigidity (17.9%), headache (50.4%), scalp allergy (10.4%), tinnitus (40.5%), alopecia (39.8%), poliosis (36.6%) and vitiligo (21.5%).

### Association of the TRAF5 and TRAF3IP2 gene polymorphisms with susceptibility to ocular Behçet's disease and VKH syndrome

Significant differences between BD patients and controls were observed for four SNPs. The frequencies of the rs12569232/TRAF5 CG genotype and C allele were significantly lower in BD patients (p_c_ = 1.08×10^−3^, OR = 0.617; p_c_ = 1.30×10^−3^, OR = 0.674, respectively), whereas a higher frequency of the GG genotype was observed (p_c_ = 1.40×10^−3^, OR = 1.583) compared with controls. As to rs10863888/TRAF5, an increased frequency of the AG genotype was observed in BD patients (p_c_ = 0.009, OR = 1.343), while a decreased GG frequency (p_c_ = 0.027, OR 0.769) was found. The frequency of the AG genotype and A allele of rs6540679/TRAF5 were remarkably increased (p_c_ = 3.6×10^−9^, OR = 1.750; p_c_ =  1.16×10^−10^, OR = 1.589 respectively), while the frequency of the homozygous GG genotype was markedly decreased (p_c_ = 1.22×10^−11^, OR = 0.536) in BD patients.

The frequencies of the AA genotype and A allele of rs13210247/TRAF3IP2 were significantly lower in BD patients (p_c_ = 1.61×10^−7^, OR = 0.391; p_c_ = 2.07×^−7^, OR = 0.417 respectively) while the frequency of the AG genotype and G allele was higher compared with controls (p_c_ = 7.05×10^−8^, OR = 2.626; p_c_ = 2.07×10^−7^, OR = 2.396 respectively) (Table 1).

Although obvious differences were found for the rs10863888 TRAF5 genotypes in VKH patients compared with healthy controls, they became non-significant after Bonferroni correction. The frequency of the CG genotype and C allele of rs12569232/TRAF5 was markedly lower in VKH patients as compared to controls. The frequency of the GG genotype was much higher (p_c_ = 1.33×10^−9^, OR = 0.446;. p_c_ = 9.45×10^−12^, OR = 0.442; p_c_ = 5.65×10^−11^, OR = 2.339), whereas the AA genotype and A allele of rs6540679/TRAF5 were both decreased in VKH patients (p_c_ = 2.61×10^−3^, OR = 0.411; p_c_ = 0.015, OR = 0.810).

As to rs13210247/TRAF3IP2, a decreased frequency of the AA genotype and A allele was observed (p_c_ = 3.85×10^−5^, OR = 0.465; p_c_ = 2.16×10^−5^, OR = 0.484) while the AG genotype was increased in VKH patients compared with normal controls (p_c_ = 3.31×10^−5^, OR = 2.177) (Table 2).

### Relationship among genotypes, gene expression at the mRNA level and downstream inflammatory factors

**Figure 2:**
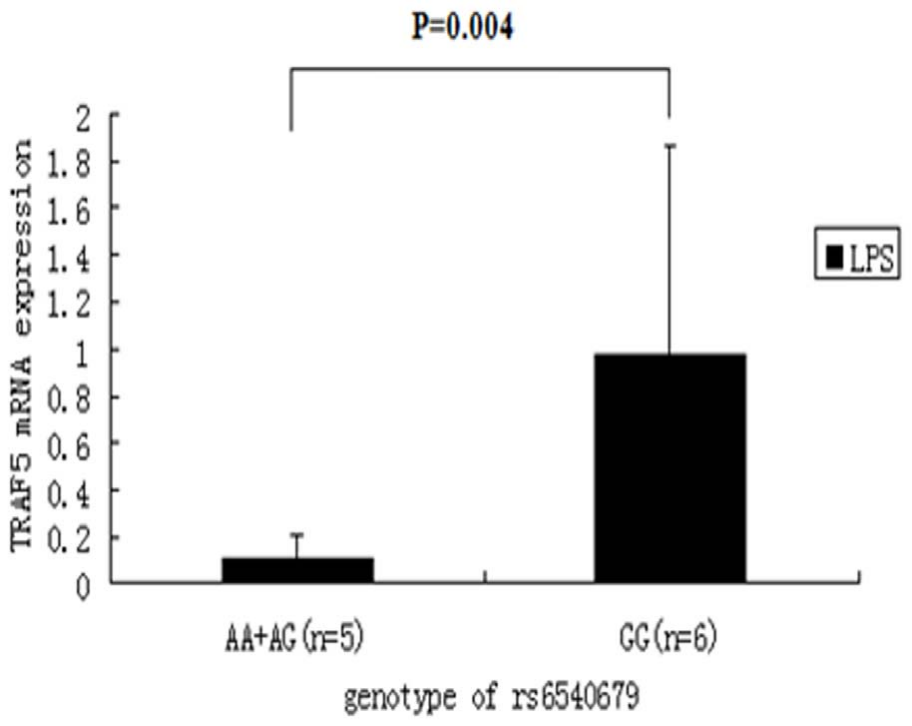
Effect of TRAF5 rs6540679 genotypes on mRNA expression by PBMCs stimulated with LPS (1 ng/ml). The mean ± SD is given for each genotype.

Since the most significant association between TRAF gene polymorphisms and uveitis was found for TRAF5, we studied the effect of the different genotypes on the expression of TRAF5 under normal or inflammatory conditions. Real-Time PCR was performed to detect mRNA expression from PBMCs obtained from healthy controls. We genotyped 79 controls for the three SNPs of TRAF5 and then used Real-Time PCR to detect their TRAF5 expression at the mRNA level without stimulation. No difference could be detected under this condition (Figure S1). Following stimulation by LPS, carriers with the GG genotype in SNP rs6540679 had a higher TRAF5 mRNA expression compared with individuals carrying the AA and AG genotype (p = 0.004) (Figure 2). No effect on TRAF5 mRNA expression was observed for rs12569232 or rs10863888 (data not shown). Furthermore no effect on TRAF5 gene expression was observed when PBMCs were stimulated by a cocktail of anti-CD3/CD28 antibodies.

**Figure 3:**
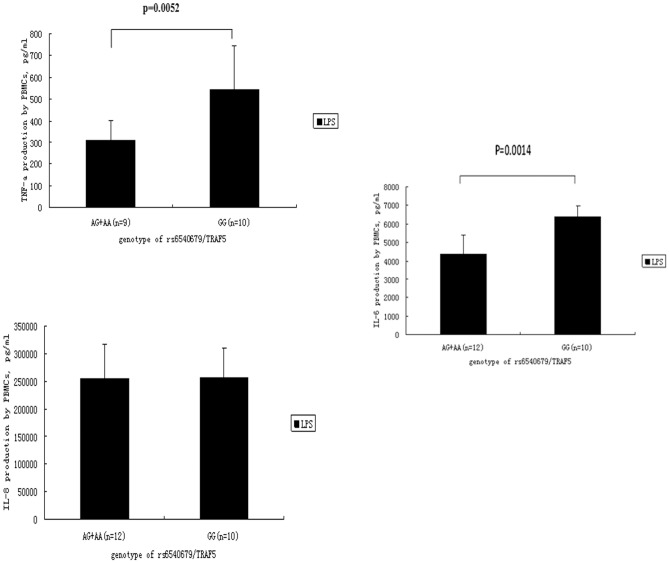
The effect of different genotypes of SNP rs6540679/TRAF5 on LPS-stimulated cytokine secretion by PBMCs obtained from healthy individuals. Supernatants of PBMCs co-cultured with 1 ng/mL LPS for 72 h were used to determine TRAF5 downstream cytokines (including TNF-α, IL-8, IL-6) levels by ELISA. Data are presented as means±SD.

The aforementioned result showed that different genotypes of rs6540679/TRAF5 could affect TRAF5 expression and therefore a further study was designed to investigate if different genotypes of rs6540679/TRAF5 could also affect the cytokine response of PBMCs following LPS stimulation. We measured the level of TNF-α, IL-6 and IL-8 (n = 22), which are important TRAF5 downstream factors [31], [32], [33], [34], [35] in PBMC cultured supernatants by ELISA. Carriers of the GG genotype showed a higher TNF-α and IL-6 secretion, compared with AA and AG carriers (p = 0.0052, and p = 0.0014 respectively) (Figure 3). No effect of the various TRAF5 rs6540679 genotypes on IL-8 production could be detected.

## Discussion

In this study, we show that TRAF5 and TRAF3IP2 gene polymorphisms are associated with VKH syndrome and ocular BD in a Han Chinese population. Highest p values were observed for the association between uveitis and TRAF5, and we therefore focused on this SNP to investigate a possible functional association. This approach revealed that carriers of the GG genotype in SNP rs6540679/TRAF5 had a higher TRAF5 mRNA expression level and enhanced TNF-α and IL-6 secretion, compared to AA and AG carriers. Expression was not altered for the other TRAF5 SNPs. We did not perform a haplotype analysis since the sample size was too small.

Our finding that the G allele of TRAF5 rs6540679 had a protective effect (OR = 0.629) in BD and a risk effect (OR = 1.235) in VKH suggests that the role of these factors depends on the context whereby they are involved during the process of inflammation. Similarly TRAF rs12569232 is most significant in VKH while rs6540679 is the most significant in BD. The functional studies performed until now have only addressed the mRNA expression and as yet no studies have addressed the effect of the gene polymorphisms on the binding characteristics of the factors and their final effect on for instance NF-kappa-B activation. Both uveitis entities we investigated in this study differ markedly as to the causative mechanisms and the role of TRAF family members for each disease might be different. BD is considered to be caused by an aberrant inflammatory response towards certain environmental triggers whereas VKH is an autoimmune disease directed against melanocyte antigens [36], [37]. How the members of the TRAF family exactly influence the inflammatory response in these two diseases remains to be elucidated.

Taken together, the results confirm earlier studies showing a role for TRAF5 in the control of inflammatory responses and the increased risk of certain polymorphisms of this gene for autoimmune or autoinflammatory diseases (Table 3) [17], [18], [32], [33], [34]. Of the TRAF5 polymorphisms included in our study, only rs10863888 was included in earlier studies on the association with RA. Associations with the TRAF5 SNPs rs6540679 and rs12569232 have not yet been reported earlier. On the other hand we did not include TRAF5 rs7514863, which showed an association with RA, because we designed six PCR primers of this SNP for PCR-RFLP, but none of their PCR product was satisfactory to be included in a following digestion step. In addition, no TaqMan® SNP Genotyping Assay is available for rs7514863.

**Table 3:** TRAF family gene polymorphisms in autoimmune diseases.

Gene	SNP	Autoimmune	Race	P value	OR (95%CI)
		Disease			
TRAF1 [51], [52], [53]	rs3761847	SLE	Japanese	0.016	1.36 (1.05–1.76)
		RA	North American	4×10^−14^	1.30 (1.23–1.42)
			& Swedish		
		RA	Japanese	0.031	1.18 (1.01–1.39)
	rs7021206	RA	Korean	0.0037	1.21 (1.06–1.38)
TRAF6 [54]	rs5030445	SLE	African American	0.00173	0.68 (0.53–0.87)
	rs5030437	SLE	African American	0.004456	0.70 (0.54–0.90)
	rs5030472	SLE	European	0.003342	0.65 (0.49–0.87)
	rs5030470	SLE	European	0.000179	0.55 (0.40–0.76)
TRAF3IP2 [23], [24], [55], [56]	rs33980500	psoriasis	German	1.24×10^−16^	*
		PsA	German	4.57×10^−12^	1.95
		SLE	Italian	0.021	1.71
	rs13210247	psoriasis	German	2..36×10^−10^	*
		PsA	German	5.76×10^−7^	2.08 (1.61–2.69)
		BD	Chinese	7.05×10^−8^	2.63 (1.87–3.68)
		VKH	Chinese	3.31×10^−5^	2.18 (1.56–3.05)
	rs13190932	PsA	German	9.19×10^−7^	2.19 (1.66–2.88)
		UC	Italian	0.02	5.05 (1.12–2.83)
		CD	Italian	0.026	3.03 (1.1–8.3)
	rs13196377	PsA	German	9.36×10^−7^	2.17 (1.66–2.88)
		SLE	Italian	0.002	2.59 (1.39–4.80)
		UC	Italian	0.049	4.1 (0.91–18.18)
TRAF5 [18]	rs10863888	RA	UK Caucasian	0.003	1.39 (1.11–1.75)
		BD	Chinese	0.009	1.34 (1.13–1.60)
	rs7514863	RA	UK Caucasian	0.005	1.2 (1.06–1.36)

The data not stated in paper.

SLE, Systemic Lupus Erythematosus; RA, Rheumatoid Arthritis; PsA, Psoriatic Arthritis; UC, Ulcerative Colitis; CD, Crohn's Disease.

VKH syndrome and BD are two of the most common uveitis entities in China [38], [39]. Both diseases account for approximately one-third of the uveitis prevalence in our country [39]. The genetic background of both diseases is not yet completely clear. In recent years, we have reported on the association of various immune response related genes with the susceptibility to both BD and VKH including STAT4, STAT3, JAK2, CD40 [7], [8], [9], [40]. In the present study, the selection of the candidate locus/gene was primarily based on GWAS data in patients with psoriasis, psoriatic arthritis (PsA) [24] and psoriasis vulgaris (PsV) [23], previously described associations for inflammatory diseases [17], [25], [41], [42], as well as the involvement of the gene products in the control of the immune and inflammatory response [43], [44], [45].

TRAF5 and Act1, encoded by the TRAF3IP2 gene, have been implicated in the control of immune and inflammatory responses [44], [45], [46]. Both proteins act as regulators of NF-kappa-B activation. During recent years, evidence is mounting that they are involved in the pathogenesis of various autoimmune and autoinflammatory disorders [17], [32], [33], [42], [47]. In view of the fact that inflammation can induce Act1-TRAF2-TRAF5 complex formation, and in turn, can stimulate a downstream stress reaction, we aimed to assess whether these two genes were involved in the susceptibility to develop BD and VKH syndrome. The polymorphisms we used were based on earlier reports showing significant associations with RA, psoriatic arthritis, psoriasis and Inflammatory Bowel Disease [18], [23], [24], [25] and included three SNPs (rs6540679, rs12569232, rs10863888) of TRAF5 and three SNPs (rs13210247, rs33980500, rs13190932) of TRAF3IP2 (Table 3).

To ensure the validity of our data we took the following measures. First, the sample sizes of VKH patients, BD patients and normal controls were large enough to ensure an association analysis, and our sample was larger than previous reports [7], [8], [18], [25], [32], [48], [49], [50]. Second, the controls were strictly selected according to their birth origin to obtain a comparable immunogenetic background. Additionally, we randomly selected 10% of the samples to undergo direct sequencing to validate the results of genotyping by PCR-RFLP.

Although our results suggested that TRAF5 and TRAF3IP2 are associated with the development of BD and VKH syndrome, it is still unknown how these SNPs exert their roles in these two diseases. The complex and redundant function of the members of the TRAF family and the context they are involved in may dictate their possible divergent roles in immune mediated diseases.

Taken together, our study, for the first time, provides evidence for a role of TRAF5 and TRAF3IP2 polymorphisms in the development of BD and VKH syndrome.

## Supporting Information

Table S1 **Clinical features of the investigated BD patients.**
(DOC)

Table S2 **Clinical features of the investigated VKH patients.**
(DOC)

Table S3 **Primers and restriction enzymes used for RFLP analysis of the TRAF5 and TRAF3IP2 gene polymorphisms.**
(DOC)

Table S4 **RT-PCR primers for genes.**
(DOC)

Figure S1 **TRAF5 mRNA expression levels in PBMCs without stimulation, according to the genotypes of the three SNPs (rs6540679, rs10863888, rs12569232 respectively).** Data are presented as means ± SD.
(TIF)
