# Supplementary material for: TRAF5 and TRAF3IP2 Gene Polymorphisms Are Associated with Behçet's Disease and Vogt-Koyanagi-Harada Syndrome: A Case-Control Study
Source: PLoS One. 2014 Jan 8;9(1):e84214. doi: 10.1371/journal.pone.0084214 (PMC3885545; doi:10.1371/journal.pone.0084214)
Supplement: Table S1 — Clinical features of the investigated BD patients. (DOC) [file pone.0084214.s001.doc]

Table S1. Clinical features of the investigated BD patients

| Clinical features | Patients with BD | |
| --- | --- | --- |
|  | N (total=789) | % |
| age at onset (years±SD) | 31.3±7.2 |  |
| Male | 579 | 73.4% |
| Female | 210 | 26.6% |
| Uveitis | 789 | 100.0% |
| oral ulcer | 789 | 100.0% |
| skin lesions | 615 | 78.0% |
| genital ulcer | 457 | 57.9% |
| Arthritis | 308 | 39.1% |
| positive pathergy test | 193 | 24.5% |
| Hypopyon | 176 | 22.31% |
