# Supplementary material for: TRAF5 and TRAF3IP2 Gene Polymorphisms Are Associated with Behçet's Disease and Vogt-Koyanagi-Harada Syndrome: A Case-Control Study
Source: PLoS One. 2014 Jan 8;9(1):e84214. doi: 10.1371/journal.pone.0084214 (PMC3885545; doi:10.1371/journal.pone.0084214)
Supplement: Table S2 — Clinical features of the investigated VKH patients. (DOC) [file pone.0084214.s002.doc]

Table S2. Clinical features of the investigated VKH patients

| Clinical features | Patients with VKH syndrome | |
| --- | --- | --- |
|  | N (total=940) | % |
| age at onset (years±SD) | 34.7±9.9 |  |
| Male | 500 | 53.2% |
| Female | 440 | 46.8% |
| Uveitis | 940 | 100.0% |
| nuchal rigidity | 168 | 17.9% |
| Headache | 474 | 50.4% |
| scalp allergy | 98 | 10.4% |
| Tinnitus | 381 | 40.5% |
| alopecia | 374 | 39.8% |
| gray hair | 344 | 36.6% |
| Vitiligo | 202 | 21.5% |
