# Supplementary material for: TRAF5 and TRAF3IP2 Gene Polymorphisms Are Associated with Behçet's Disease and Vogt-Koyanagi-Harada Syndrome: A Case-Control Study
Source: PLoS One. 2014 Jan 8;9(1):e84214. doi: 10.1371/journal.pone.0084214 (PMC3885545; doi:10.1371/journal.pone.0084214)
Supplement: Table S3 — Primers and restriction enzymes used for RFLP analysis of the TRAF5 and TRAF3IP2 gene polymorphisms. (DOC) [file pone.0084214.s003.doc]

Table S3. Primers and restriction enzymes used for RFLP analysis of the TRAF5 and TRAF3IP2 gene polymorphisms

| Gene | SNP ID | Primers | Restriction enzyme |
| --- | --- | --- | --- |
| TRAF5 | rs6540679 | 5'TGCCCTTTTTTGCTCTCCTAA 3' | AccIE* |
|  |  | 5'CCAAGGGAAGGAAACATATGAA 3' |  |
| TRAF3IP2 | rs33980500 | 5'GGGCTCCAACCACAGACT 3' | Msel* |
|  |  | 5'GTGAGGACTCCAAGAATTTATC 3' |  |
|  | rs13190932 | 5'AGGGCTCCAACCACAGACTCAG 3' | Fnu4HI* |
|  |  | 5'TGCCTGGAGGATAAGTAAACAAT 3' |  |
|  | rs13210247 | 5'GGGGACAACTCTAAGGCAAGGA 3' | MvaI* |
|  |  | 5'GACGCTTCAAACCTATTCCTG 3' |  |

* Fermentas China Co. Ltd., Shenzhen, China
