# Supplementary material for: TRAF5 and TRAF3IP2 Gene Polymorphisms Are Associated with Behçet's Disease and Vogt-Koyanagi-Harada Syndrome: A Case-Control Study
Source: PLoS One. 2014 Jan 8;9(1):e84214. doi: 10.1371/journal.pone.0084214 (PMC3885545; doi:10.1371/journal.pone.0084214)
Supplement: Table S4 — RT-PCR primers for genes. (DOC) [file pone.0084214.s004.doc]

Table S4. RT-PCR primers for genes.

| Gene | RT-PCR primers |
| --- | --- |
| TRAF5F： | 5’CGGTTCTGAAGCGGAATGGCT3’ |
|  | 5’AGCGCTCTTCCAACCGCTCC3’ |
| TRAF3IP2F | 5’GCGCTACCTTAGGGATAAGACCGT3’ |
|  | 5’ CAGCTGCGACTCAGCGCCTT3’ |
