# Supplementary figures and images for: TRAF5 and TRAF3IP2 Gene Polymorphisms Are Associated with Behçet's Disease and Vogt-Koyanagi-Harada Syndrome: A Case-Control Study
Source: PLoS One. 2014 Jan 8;9(1):e84214. doi: 10.1371/journal.pone.0084214 (PMC3885545; doi:10.1371/journal.pone.0084214)

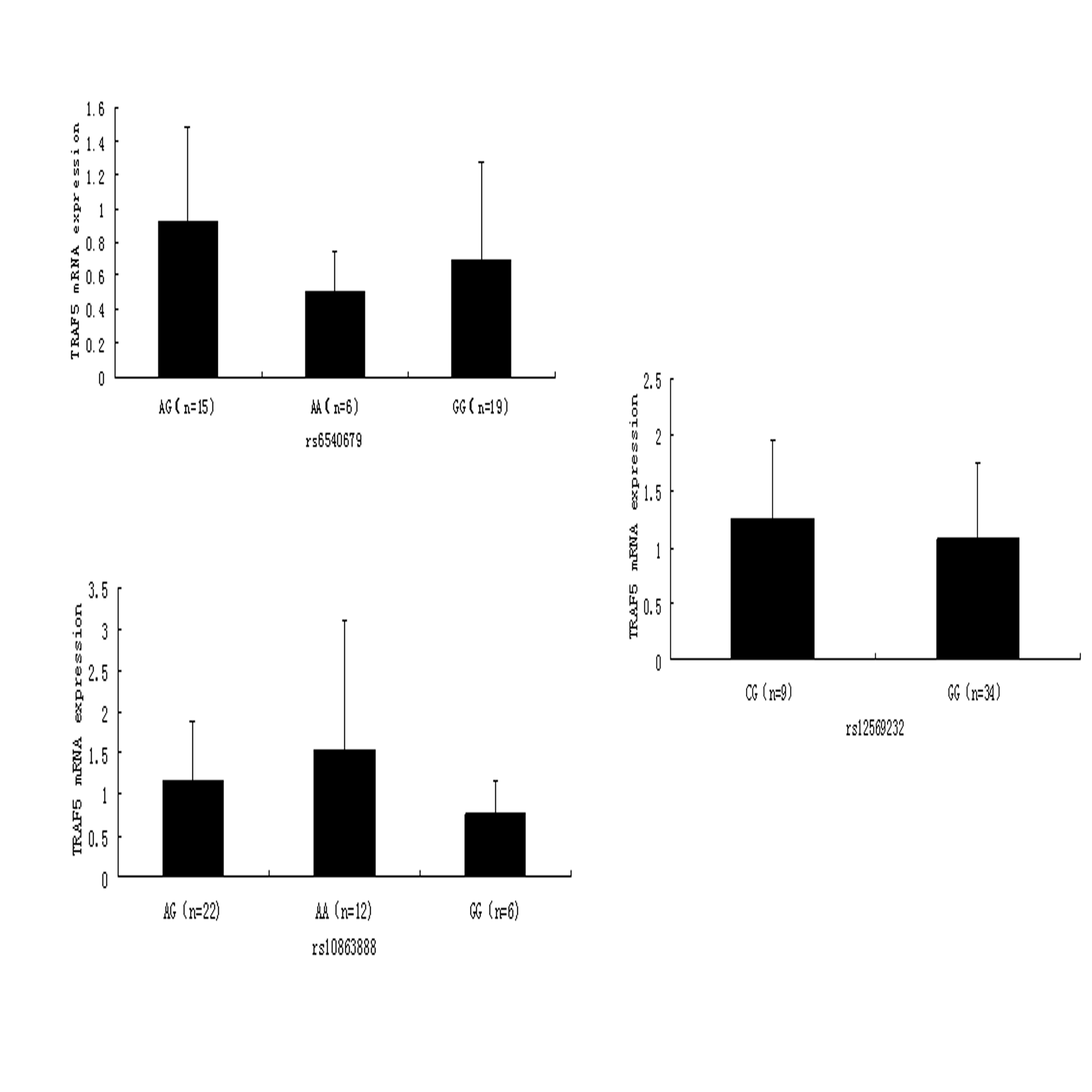

Supplement: Figure S1 — TRAF5 mRNA expression levels in PBMCs without stimulation, according to the genotypes of the three SNPs (rs6540679, rs10863888, rs12569232 respectively). Data are presented as means ± SD. (TIF) [file pone.0084214.s005.tif]
